# Understanding the interactions that children and young people have with their natural and built environments: A survey to identify targets for active travel behaviour change in Wales

**DOI:** 10.1371/journal.pone.0311498

**Published:** 2024-10-18

**Authors:** Emily Holmes, Marco Arkesteijn, Kim Knowles, Tracie McKinney, Amy Mizen, Catherine Purcell

**Affiliations:** 1 North Wales Medical School, Bangor University, Wales, United Kingdom; 2 Institute of Biological, Environmental and Rural Sciences, Aberystwyth University, Wales, United Kingdom; 3 Department of Theatre Film and Television Studies, Aberystwyth University, Wales, United Kingdom; 4 Faculty of Computing, Engineering and Science, University of South Wales, Wales, United Kingdom; 5 Environment and Health Research Centre, Swansea University Medical School, Swansea University, Wales, United Kingdom; 6 School of Healthcare Sciences, Cardiff University, Wales, United Kingdom; Nelson Mandela African Institute of Science and Technology, UNITED REPUBLIC OF TANZANIA

## Abstract

Active travel offers many societal benefits, including improving people’s mental and physical health and minimising our impacts on the environment. Increasing active travel is particularly important amongst children and young people (CYP), who are building habits which they will carry into adulthood. Studies on active travel amongst CYP are limited, however, with most research focusing on adult participants or on adult perceptions of children. This study sought to understand CYP’s interactions with the built and natural environment–and therefore their access to active travel–through the Capability, Opportunity, Motivation, Behaviour (COM-B) model. With a stakeholder group representing local government, youth organisations and active travel organisations, we co-created two bilingual questionnaires–one for young people aged 12–16 years living in Wales and the other for parents of young people aged 12–16 years living in Wales. Both questionnaires collected information on behaviour and perceived capability, opportunity and motivation of CYP to engage with their natural and built environments. The questionnaires included a discrete choice experiment (DCE), which proposed a series of binary choice questions indicating preferences based on landscape, journey time and type of travel. A total of 124 questionnaires (38 young people and 86 parents) were returned for analysis. These data indicate that CYP’s time spent outdoors is not dependent upon geography (rural/urban/suburban), season, or school holidays. There was a significant difference in capability, opportunity and motivation between parents and CYP, with parents over-estimating the psychological capability of CYP to engage outdoors. The preference data indicate that active travel is the favoured mode of transport, with both CYP and parents stating that they would increase travel time in order to travel actively. While this response is not consistent with respondent’s day-to-day travel choices, it suggests that the limitations to active travel may be psychological capability and automatic motivation, rather than a lack of opportunity.

## 1. Introduction

Active travel refers to walking, cycling, wheeling, scooting, or otherwise using one’s body to make necessary journeys (as opposed to walking/hiking for leisure) [1]. Active travel is shown to improve physical health [2] and mental wellbeing [3] and can also be an important tool in tackling the environmental crisis [4]. Despite these benefits, motorised transport continues to be the dominant travel mode internationally [5]. This is replicated in Wales, which has seen little change in the frequency and duration of walking and cycling since the Active Travel (Wales) Act was introduced in 2013 [6]. Data from the latest National Survey for Wales, conducted in March 2021, suggest that only 9% of adults cycle and 59% of adults walked for more than 10 minutes as a means of transport [7]. However, to understand when and why people choose to travel actively, we need to take a step back and consider people’s relationships with their built and natural environments, which offer both opportunities and challenges for engaging with the outdoors [8]. Walkability of neighbourhoods and availability of recreational facilities are frequently linked with positive engagement in active travel, but otherwise psychological and environmental factors influencing active travel are inconsistent across studies [9]. For example, whilst the ‘15-minute neighbourhood’ might reduce vehicular traffic, energy consumption and carbon emissions [10–12], reduce the pressure on the public health system [13] and address the first/last mile connection issue, which refers to the disconnect between public transport and an individual’s origin or destination [14], the concept has been criticised for being physically deterministic and failing to take into account the needs of different social groups and biodiversity [15].

For children and young people (CYP), active travel is an opportunity for independence and to increase time spent outdoors. However, young people currently spend less time outdoors than any other time in history [16], coinciding with the widespread use of social media and screen time, which have been associated with the growing concern about the mental health and wellbeing of CYP internationally [17] and in Wales [18]. This concern stems from previous research that demonstrates that time spent outdoors supports good mental health and wellbeing in CYP [19], therefore finding ways to increase time spent outdoors, in any capacity, is a worthwhile goal.

Active travel is relatively well studied in terms of physical health, mental wellbeing and environmental impacts. However, because most studies focus on adult participants or on adult perceptions of children [20], there is limited consistent information on active travel in CYP. One reason for this is that previous research has identified that use of children’s outdoor space is subject to parental influence [21,22]. While parents have ultimate decision-making power, even very small children have opinions on how to organise their day and older children have growing independence to make these choices for themselves [23]. Therefore, treating young people as autonomous actors in their lives will help us understand their motivations and preferences for active travel.

It is also important to focus on CYP from a behaviour change perspective, then we can implement interventions at a time in their lives when they are building healthy future habits [24]. Based on the National Survey for Wales, 47% of children and 32% of adolescents solely walked to primary and secondary school respectively [7]. Living less than one mile from the school, parents’ frequency of walking and cycling and living in an urban area have been identified as factors positively influencing active travel to both primary and secondary schools [25]. Given the well accepted health and wellbeing benefits of blue and green space [26] and the Welsh Government priority to improve CYPs mental health and wellbeing (2022), it is essential to understand the current behaviour and the perceived capability, opportunity and motivation of young people and their parents to engage with their outdoor spaces in Wales. We cannot develop effective behaviour change interventions or identify which policy changes will be effective without this understanding. Whilst previous research has identified that changes to the built environment can facilitate active travel [27] using a theory-based understanding of behaviour change is vital for designing successful interventions [28].

The Capability, Opportunity, Motivation, Behaviour (COM-B) model, the hub of the behaviour change wheel [29], provides a comprehensive framework for understanding behaviour and for designing behaviour change interventions. The COM-B conceptualises behaviour as part of a system of interacting factors. Capability can manifest as either physical attributes, such as skill and strength, or psychological attributes, including knowledge and mental resilience. Opportunity can arise from physical aspects, such as environmental conditions, time availability, or resource accessibility, as well as social factors like social norms, cues and interpersonal influences. Motivation can stem from reflective processes, such as deliberate plans or conscious intentions, as well as automatic responses, including reactions, habits, desires and impulses. According to the COM-B model, for a given behaviour to occur, at a given moment, one must have the capability and opportunity to engage in the behaviour, with the strength of motivation to engage in the behaviour greater than for any other competing behaviours [29]. The COM-B is widely used in public health research and initiatives, for example it has recently been used to understand the habits and behaviours of inactive CYP [30] and to understand the capability, opportunity and motivation of performing COVID-19 disease prevention behaviours [31]. Likewise, it has been used as a framework to determine the barriers and enablers to delivery of the Healthy Kids Check in Australia [32] and as an explanatory framework in understanding greenspace usage in southeast England [33].

In the context of active travel, the COM-B has also been used explore children’s (aged 9–10 years) experiences of their journey to school, with the authors reporting that environment context, emotions, social influences and trip factors underlie children’s affective experiences of their journeys to school [34]. Additionally, it has been utilised to examine how various elements of temporary Streetspace schemes in London serve as either obstacles or facilitators to walking and cycling for short local trips [35]. The authors found that factors related to opportunity and motivation were identified as both barriers and enablers based on 21 semi-structured interviews [35].

Alongside targets for behaviour change we can consider how CYP value different characteristics associated with travel, that may also influence their behaviour. Stated preference techniques, rooted in economic theory, offer of hypothetical method for eliciting CYP preferences for travel options without directly asking them to reveal their preferred option for a specific journey. These methods assume that peoples’ preferences are revealed though choices; choices can be described in terms of bundles of attributes (characteristics e.g., built, or natural environment); and attributes can be traded to maximise utility/satisfaction [36,37]. This framework has the potential to further our understanding of how the characteristics of a journey impact the preferences for active travel; to predict the uptake of active travel for a specific journey; and, to also identify potential differences in uptake between subgroups (stakeholders, sociodemographic characteristics and behavioural factors).

The Wales Active Travel Research Consortium (ATLAS) was formed in 2022 by an interdisciplinary group of researchers representing six Welsh Universities. Our initial project, understanding the interactions that children and young people currently have with their natural and built environments (INHABIT), was focused on understanding the motivations, barriers and opportunities for active travel (following the COM-B model) amongst young people aged 12–16 years old and their parents. From the outset, ATLAS recognised that ‘people make research’ and committed to working in partnership with local authorities, third sector organisations (e.g., Sustrans, Cycling UK and Urdd Gobaith Cymru), parents and young people, who were instrumental in shaping the project aim, data collection tools and interpretation of the findings.

The aim of this study was to understand how CYP in Wales are interacting with their built and natural environment in the context of the COM-B model of behaviour change [29]. The hypotheses were: (1) young people will interact with their built and natural environments differently depending on whether they live in suburban, urban, or rural parts of Wales, time of year and within and outside of school term times; (2) young people and parents will differ in terms of perceived capability, opportunity and motivation to spend time outdoors; (3) young people that spend more time outdoors will have higher perceived capability, opportunity and motivation compared with young people that spend less time outdoors. A secondary objective was to explore the feasibility of using stated preference methods to elicit CYP and parent preferences for travel and explore how this data could be used to predict the probability of uptake for an exemplar journey. By applying the COM-B, the study aimed to elucidate the influences shaping CYP’s behaviours in Wales in relation to their environment, thus informing future research aimed at developing interventions and policies that promote healthier and more sustainable behaviours among this demographic.

## 2. Methods

This project aligns with bounded relativist ontology [38] whereby a shared reality of built and natural environments is bounded within the cultural context of Wales, whilst acknowledging that different realities between parents and young people may exist as well as cultural subtleties between geographical boundaries. A constructionist epistemology [39] then follows, as meaning of the built and natural environments exist because of engagement with these environments and through active and symbolic language. However, theoretically these realities and meanings can be explored empirically and pragmatists believe that research should be contextually situated, without being committed to one philosophical position in order to understand what is happening at any one point in time [38]. Taking the theoretical perspective of pragmatism therefore, questionnaire methods can be used to understand how CYP in Wales are interacting with their built and natural environments in the context of the COM-B model of behaviour change [29] and to explore stated preferences for journeys using a discrete choice experiment (DCE) [40]. The aim was therefore addressed using a questionnaire data collection method and situated within the theoretical perspective of pragmatism.

### 2.1. Stakeholder engagement

We recruited a stakeholder group from a wide range of organisations such as local Government, youth organisations and active travel organisations, as well as parents and CYP. The stakeholder group met face-to-face and online to co-create two bilingual questionnaires, one for parents of 12–16-year-olds living in Wales and one for young people aged 12–16 years living in Wales. Both questionnaires intended to determine current behaviour and the perceived capability, opportunity and motivation of CYP and their parents to engage with built and natural environments. The questionnaires also gathered the following demographic information: chronological age, sex, disability status, first 3–4 letters/numbers of their postcode combination (e.g. NP18) and ethnicity. A series of binary choice questions were included asking respondents to indicate their preferences based on time, type of transport and landscape, i.e., the DCE.

**Fig 1:**
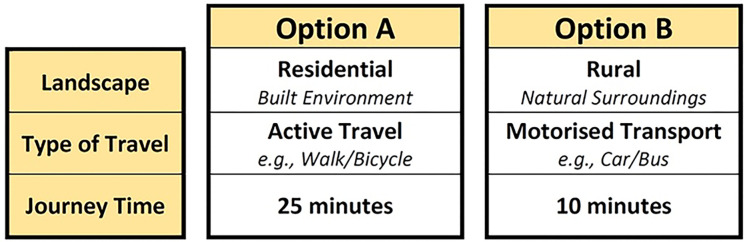
Example of one of four hypothetical choice-sets showing the two levels and three attributes, in the DCE, CYP version. Imagine you have two options of how you make a journey. Please indicate which of the following options, Option A or Option B, you would prefer. There are no right or wrong answers, we are simply interested in your views.

The DCE comprised three attributes, each with two levels, that were paired into 8 scenarios (4 binary choice sets, Fig 1) using an efficient design, generated using experimental software (Choice Metrics, 2018, Ngene Software version 1.2.1). Attributes and levels were selected by the study team and verified by stakeholder engagement. The hypothetical scenario was described as "a journey‴ to avoid a focus on travel to school, which we know to be associated with additional barriers. Wording was adjusted for CYP and parents to ensure the choice was focused on that of the CYP i.e., please indicate which option… you would prefer your child to take.

### 2.2. Survey data collection

Data collection took place between the 24^th^ April 2023 and 11^th^ September 2023. To recruit parents and CYP the study was advertised though social media and existing networks with the help of our stakeholder group. The advertisement included two links, which when accessed, directed potential participants to the relevant participant information sheet, followed by the related e-consent form. If e-consent was provided, then participants accessed the appropriate questionnaire (i.e. either parent or CYP in either English or Welsh), once a participant submitted the questionnaire, a debrief sheet became visible. Respondents could enter a prize draw by providing their (or their parents) email address in a separate online form. Ethical approval was obtained from the School of Healthcare Sciences, Cardiff University (ref: REC985) and informed consent was obtained from participating parents and CYP. Informed consent from the parents of participating CYP was not specifically required, as the included age range were deemed able to provide informed consent themselves by the ethics committee, parents were however required to provide their email address to enter the prize draw. The study was registered on Open Science Framework (10.17605/OSF.IO/H3EDP).

#### 2.2.1 Questionnaire characteristics

Both questionnaires, provided in S1 and S2 Files, were co-created with our stakeholder group. Following seven demographical questions (age, gender, ethnicity, additional learning needs, type of active travel mode owned, home postcode, property type), parents and CYP were asked to respond to a series of questions relating to their interactions with outdoor space. The built environment was assessed according to the rural or urban characteristics of participants, which were determined from postcode data. We provided a definition of outdoor space, as any public space that is outside excluding private gardens. There were 28 questions in total that explored natural and built environment attributes in the context of capability, opportunity and motivation. Specifically, five relating to physical capability four on psychological capability, six on reflective motivation, five on automatic motivation, four on physical opportunity and four on social opportunity. All questions required a response on a five-point Likert scale between ‘strongly agree’ and ‘strongly disagree’. Following these questions, four questions explored behaviour by asking respondents to indicate typical hours spent outdoors during winter and summer months and school term or holiday periods. This was followed by four binary choice questions asking respondents to indicate their preferences based on time, type of transport and landscape (the DCE).

### 2.3. Data analysis

For all inferential statistics assumption tests were conducted prior to running the inferential test and appropriate changes made if any of the assumptions were violated. Initially we conducted Cronbach’s Alpha reliability analysis on each construct of the COM-B to determine whether it was appropriate to reduce the data into those constructs (six constructs: physical capability; psychological capability; psychological opportunity; social opportunity; automatic motivation and reflective motivation) or into fewer constructs (three constructs: capability, opportunity, motivation). We conducted descriptive statistics on all the variables described above and highlight any specific areas within each COM-B construct that were particularly high/low. Where descriptive data suggested differences between COM-B components, we conducted two-way ANOVAs to explore whether these differences in COM-B components between young people and parents were statistically significant. We conducted correlations between each COM-B construct for parents and young people. Finally, we conducted regression analyses for parents and young people to determine which factors influence behaviour from: COM-B components, landscape (e.g. suburban, urban or rural), gender, disability status and ethnicity.

Stated preference data was analysed using random effects logit regression models that allow for multiple choices per respondent, to identify the significance, direction and magnitude of preferences. Confidence intervals were generated using 1000 bootstrap replications. Subgroups were predefined as: (1) Population: CYP or parent, (2) Age: older or younger; (3) Sex: male or female; (4) Welsh Index of Multiple Deprivation (WIMD), the Welsh Government’s official measure of relative deprivation for small areas in Wales: higher or lower; (5) Screen time: higher or lower; (6) COM-B scores; and restricted to n≥30 per model, then tested using likelihood-ratio tests. To illustrate how the preferences elicited using the DCE could be used to model expected utility and probability of uptake of different journeys, an exemplar was selected by the study team (EH/CP) to illustrate four realistic journey options. The exemplar represents a recreational journey from a residential area of Newport, South Wales, UK, to the local shopping centre. The expected utility and probability of each option were calculated by combining the preference weights for landscape, travel and time (derived from the regression of DCE survey data), with data obtained from Google Maps (https://www.google.com/maps; accessed 05/10/2023) based on a starting point, end point and travel modes. Parameters are specified in Table 1. Model specifications and calculations are detailed in S3 File. Preference analyses were completed in STATA 18 and Microsoft Excel.

**Table 1:** Parameters used in the utility model to estimate probability of uptake for exemplar journey.

Attribute (coding)	Walk	Cycle	Car	Bus
Landscape				
Rural (1)	0.25	0.25	0.00	0.00
Residential (0)	0.75	0.75	1.00	1.00
Type of travel				
Active (1)	1.00	1.00	0.00	0.00
Motorised (0)	0.00	0.00	1.00	1.00
Time (minutes)	42.00	13.00	10.00	22.00

## 3. Results

### 3.1. Demographic information

**Fig 2:**
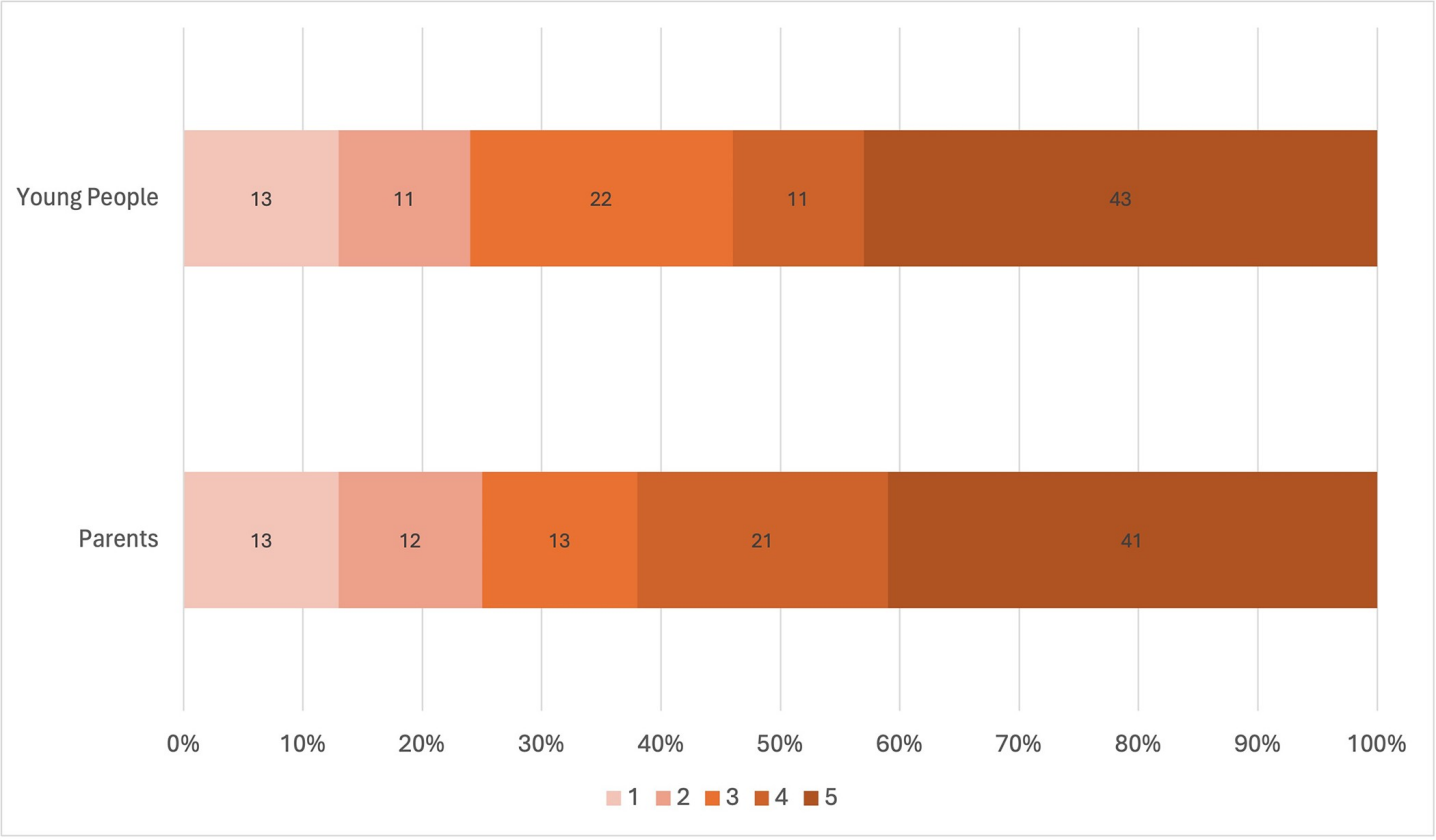
Parent and young people quintiles of Welsh Index of Multiple Deprivation. Values are expressed as a percentage, with blue indicating the most deprived area and orange the least deprived.

The combined sample (N = 124) was made up of 38 young people and 86 parents. Fig 2 shows the WIMD for the parent and CYP respondents.

#### 3.1.1. Parents

As shown in Table 2 the parent sample completed the parent questionnaire for 53 male (62%), 32 female (37%) and one other (1%) young people with a mean age of 13.70 years (SD = 1.40). Of these, 72 were classified as white (84%), two preferred not to say (2%) and 12 were of another (14%) ethnicity. A total of 68 parents reported that their children did not have an additional learning need (79%), with 18 reporting an additional learning need (21%). A total of 58 parents reported that their children lived in an urban landscape (67%), 25 reported a rural landscape (29%) and one a suburban landscape (2%).

**Table 2:** Demographic information for parent and children and young people respondents.

	Parents	Children and young people
Gender		
Male	53	16
Female	32	20
Other	1	2
Ethnicity		
White	72	27
Preferred not to say	2	1
Another	12	10
Additional Learning Needs	18	8
Landscape		
Urban	58	30
Rural	25	7
Suburban	1	0

#### 3.1.2. Children and young people

As shown in Table 2 the CYP questionnaire responses represented 16 males (42%), 20 females (53%) and two who preferred not to say (5%). Of these, 27 classified themselves as white (71%), one preferred not to say (3%) and 10 selected another (26%) ethnicity. A total of 30 self-reported as not having an additional learning need (79%), with 8 reporting an additional learning need (21%). A total of 30 young people reported living in an urban landscape (79%), seven a rural landscape (18%) and one participant did not respond to this question (3%).

It is worth highlighting the comparison between our sample characteristics and the Welsh population. Firstly, while the population of Wales is predominantly white at 95% [41], our survey was completed by 84% of parents and 71% of children and young people who identified as white. Additionally, the percentage of individuals in Wales with additional learning needs is reported to be 13.4% [41], whereas in our sample, 21% of children and young people self-reported having an additional learning need. These differences suggest that we successfully recruited representation from some underserved groups. Furthermore, while the population of Wales is 49% male [42], our sample consisted of 62% male parents and 42% male children and young people. Finally, approximately 80% of the Welsh population live in urban areas, compared to 73% in our sample.

### 3.2. Interactions with built and natural environments

A Cronbach’s Alpha was initially conducted on all 28 items, with results indicating a high (very reliable) level of internal consistency (.87). This suggests that the items could be reduced to the six components of the COM-B (physical capability, psychological capability, physical opportunity, social opportunity, reflective motivation and automatic motivation). A Cronbach’s Alpha on these six constructs (COM) revealed an acceptable (reliable) level of internal consistency (.78) which would not be improved by deleting any of the items. Finally, a Cronbach’s Alpha was conducted on three constructs (capability, opportunity and motivation), which revealed a questionable (reliable) level of internal consistency (.68). This could have been improved slightly by removing capability (.70) but this was not deemed to make a sufficient difference to justify its removal.

Our first hypothesis was that young people would interact with their built and natural environments differently depending on whether they live in suburban, urban or rural parts of Wales, time of year and within and outside of school term times. A two-way ANOVA with geography [suburban, urban and rural] and hours spent outdoors in winter and summer term times and school holidays revealed no significant differences.

### 3.3. COM-B

Our second hypothesis was that young people and parents would differ in terms of perceived capability, opportunity and motivation to spend time outdoors. A two-way ANOVA with COM components [capability, opportunity, motivation] and group [parents, children] revealed a significant difference in psychological capability between parents and children (*F*(1,122) = 5.68, *p* = .02), with parents reporting that their children had significantly greater psychological capability compared to the self-reports of CYP. No other significant differences were found.

Our third hypothesis was that CYP who report spending more time outdoors would have higher perceived capability, opportunity and motivation compared with CYP that spend less time outdoors. A Pearson correlation revealed a small positive correlation between capability and the number of hours spent outdoors in a normal week (*r*_*p*_(.35, *p* = .02), in winter term time (*r*_*p*_(.36, *p* = .01), winter school holidays (*r*_*p*_(.29, *p* = .04), summer term time (*r*_*p*_(.29, *p* = .04) and summer school holidays (*r*_*p*_(.32, *p* = .03). No significant correlations were found for opportunity or motivation. However, a multiple linear regression was run to predict time spent outdoors in a normal week, capability, opportunity and motivation. These variables statistically predicted, albeit a low prediction, time spent outdoors (*F*(3,120) = 5.57, *p* = .001, *R*^*2*^ = .12. Two of the three variables added statistically significantly to the prediction, capability = .04 and motivation, = .03. When looking at capability and motivation in more depth through a multiple linear regression to predict time spent outdoors in a normal week, physical capability, psychological capability, automatic motivation and reflective motivation, it was found that automatic motivation significantly predicted time spent outdoors = .02.

### 3.4. Preference elicitation

Our secondary objective was to explore the feasibility of using stated preference methods to elicit CYP and parent preferences for travel and explore how this data could be used to predict the probability of uptake for an exemplar journey. The DCE was completed by 124 respondents (38 young people, 86 parents) resulting in 992 observations. Likelihood ratio testing indicated that the restricted model (combined population) was different to that of an unrestricted model (CYP, parents) and therefore the unrestricted model (CYP, parents) provided our base case (Table 3).

**Table 3:** Results of the DCE random-effects logistic regression model.

	β co-efficient	p-value	95% Confidence Interval		MRS	95% Confidence Interval	
**CYP**							
Landscape	0.8207	0.0010	0.3738	1.4092	20.7840	7.1577	81.7767
Type of travel	1.1440	<0.0001	0.7016	1.6870	28.9727	12.4054	126.3999
Time	-0.0395	0.0160	-0.0754	-0.0079			
Constant	-0.2913	0.4030	-1.0129	0.4023			
No. of observations	304						
No. of groups	38						
Log likelihood	-191.53						
**Parents**							
Landscape	0.6811	<0.0001	0.2437	1.054188	19.0040	6.3066	51.6883
Type of travel	2.7302	<0.0001	2.4059	3.241882	76.1733	46.4178	196.9248
Time	-0.0358	0.0050	-0.0620	-.0138525			
Constant	-1.0784	<0.0001	-1.6189	-0.5754			
No. of observations	688						
No. of groups	86						
Log likelihood	-345.26						

Subgroup analyses were restricted to groups within parent responses, due to sample size, and indicated their preferences vary by (3) Sex of the CYP: male or female (*p* = 0.0044); and (6) COM-B scores on total motivation (*p* = 0.0003). Exploratory analysis on motivation, also revealed significant differences in preferences accounting for reflective (*p* = 0.0083) and automatic (*p* = 0.0003) motivation. There were no significant differences in models accounting for: (2) Age of CYP: older or younger; (4) WIMD: higher or lower; (5) Screen time: higher or lower; and (6) COM-B scores on total capability or total opportunity.

**Fig 3:**
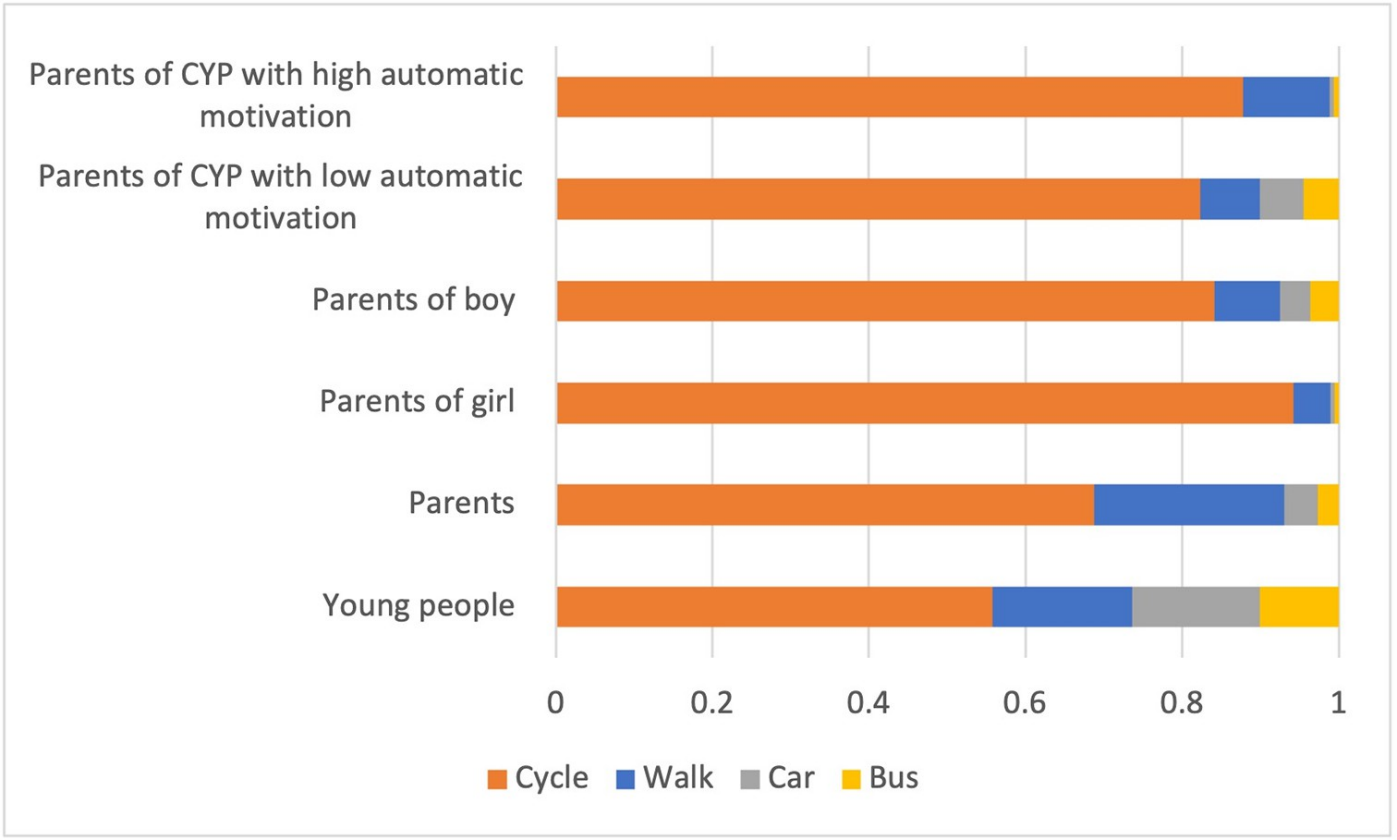
Probability of uptake of mode of transport by subgroup. CYP = Children and Young People.

When preference weights elicited from the DCEs were applied to the exemplar of travelling from a residential area of Newport to the local shopping centre, active travel dominated (cycle or walk) with most preferred mode of transport across subgroups being bicycle (Fig 3). The proportion of motorised transport is highest for CYP, but lowest when parents report children as having high automatic motivation.

## 4. Discussion

The present study provides initial evidence that CYPs behaviour towards their built and natural environments are not influenced by rurality, or time of year. The DCE completion rate suggests this is an acceptable method for eliciting preferences of CYP and parents and was able to show a strong effect of parental motivation on behavioural preferences for their child. Both physical and psychological capability correlated positively with time spend outdoors, but primarily showed that parents overestimated the psychological capability of CYP. Based on the linear regression, opportunity was not found to influence behaviour. Automatic motivation rather than reflective motivation might play a role in CYPs time spent outdoors, whilst both motivation types played a primary role in parental behavioural decision making/preferences.

Overall, there were no significant differences between parents’ perception of their children and young people’s own perceptions in relation to their interactions with their built and natural environments. This is consistent with previous research which has found that parents attitudes towards natural environments are related to the amount of time children spent outdoors [43]. Previous research during Covid-19 identified that physical opportunity and reflective motivation predicted physical activity behaviour amongst 1,521 UK adults [44]. Interestingly, in a recent systematic review which included 33 peer-reviewed articles relating to children’s (aged 4–12 years) outdoor play, they identified individual and environmental correlates of outdoor play, concluding that there is a need to promote children’s capability and motivation and provide them with social and physical opportunities [45]. Therefore, it appears that high levels of motivation, physical opportunity and social opportunity to spend time in natural environments are needed. The present study however suggests that when it comes to spending time outdoors the difference between automatic and reflective motivation should be considered. This is consistent with previous research that identified emotions relating to perceived environmental and personal safety were important in children’s experiences of their journey to school [34] and a qualitative study that found that motivation was both a barrier and enabler to walking and cycling for short trips [35]. Given that automatic motivation is considered less susceptible to behaviour change, this might pose a barrier in persuading CYP and their parents to spend more time outdoors.

Furthermore, a longitudinal study in Australia found that individual and social factors predict children’s time outdoors [46]. In addition, our findings suggest that motivation plays an important role in CYP spending time outdoors. In a study exploring what children aged between 9–10 years liked and disliked about their journey to school, in five primary schools in Newcastle upon Tyne, UK, the authors suggest that commuting to school may be related to other activities that children enjoy, whereby children who prefer car journeys to school may also enjoy more passive activities [34]. The authors suggest that further exploration is needed to explore whether a supportive built environment for active travel to school could shape children’s reflexive motivation for future trips [34]. It is possible that the data from our small-scale study mirrors this, the CYP and parents of CYP may preferentially enjoy spending time outdoors and therefore those with high automatic motivation reported a preference for non-motorised transport.

Although not statistically different, potentially due to the small sample size, we did find that CYP spent more time outdoors in summer school holidays apart from when doing activities organised by someone else where there was little change across seasons/term and non-term time.

The behavioural preferences showed a strong effect of parental motivation on decision making and has been identified previously as a key factor in active travel for children, such as the school journey [47,48]. The DCE analysis demonstrated how preference data could be used to predict the probability of uptake for journeys and to assess the influence of both behavioural and journey attributes such as landscape, type of travel and duration. Interestingly, CYP and parents preferred CYP to travel in rural landscapes, use active travel (walk/bicycle) and have quicker journeys. one explanation for these differences could be that the majority of our sample lived in urban areas and although our study demonstrates that it was possible to determine how preference data could be combined with behavioural data and journey parameters, further research on the perception of rural landscapes is required with a larger sample. The preference for the mode of transport being active travel is strong, as suggested by a willingness to increase travel time by 30 and 76 minutes respectively for CYP and parents. Although this was greater than we anticipated, it indicates potential for longer active journey durations if other barriers are removed to promote active travel.

### 4.1. Strengths and limitations

This is the first study, to our knowledge, to attempt to examine the way in which children and young people and parents of children and young people in Wales interact with their built and natural environments using a well-established behaviour change model. It also contributes initial data, albeit on a small scale, to a growing body of research that captures the voice of children and young people, compared to research on active travel that has historically been limited to capturing the voice of parents [49]. Possibly the greatest strength of this study was the inclusion of a range of stakeholders working alongside an interdisciplinary research team in the design of the study and study materials. There are however limitations, recruitment was challenging, this may reflect a level of survey fatigue which has been reported in other disciplines, such as neurosurgery research [50], since Covid-19. Due to the limited sample size, it is important to interpret the findings cautiously. Furthermore, due to our recruitment approach self-selection bias make it difficult to generalise patterns to the whole population, however this does not negate the within sample findings connecting behaviour with preference. Our parent/child questionnaires were also not linked, meaning parents and children are not necessarily from the same family and we did not attempt to establish the presence of child-parent dyads, but rather ascertain the perspectives of, and conduct preliminary investigations on, each population. We also acknowledge that more in depth formative work would be needed prior to drawing firm conclusions from the DCE, for the purposes of this study a pragmatic approach was taken for the purpose of assessing the feasibility of using this method in future studies.

### 4.2. Implications and future directions

The present study highlights the complexity, but also the potential value of applying the COM-B to promote behaviour change in CYP and parents. Although the explained variance by the COM-B is low/moderate to predict time spent outdoors, insight that in theory people are prepared to travel longer durations, but don’t in practice, is valuable. The COM-B indicates this might be due to (psychological) capability and (automatic) motivation, rather than not having the opportunity. Removing barriers, such as increasing safety and confidence, might increase perceived capability and motivation. Psychological capability refers to one’s own perception of possessing the necessary mental skills, such as decision making, to behave in a particular way, perceiving active travel as unsafe or lacking confidence in high-traffic areas may therefore play a role [51]. In contrast, opportunity appeared to be less important, although our results from the DCE indicate increased green space can increase the likelihood of active travel, which is consistent with previous research [52]. However, it remains unclear whether it is green space that appears important, or the perception that greener routes are safer routes. Beyond this study, our public engagement activities have identified that safety bottlenecks (particular parts of an active travel route) are key in decision making between active or motorised travel.

The behavioural preference to increase time spent outdoors by travelling actively suggests there is potential for behaviour change. This finding, although tentative at this stage, is encouraging as previous research has suggested that active travel is a potential means of increasing physical activity, with active travel to non-school destinations associated with higher overall physical activity levels in children aged 9–10 years [53,54]. This is important as prolonged sedentary behaviour has been identified as a health risk factor in children, which in combination with obesity can lead to chronic diseases later in life [55]. Therefore instilling physical activity and limiting sedentary habits in childhood is crucial as these behaviours tend to persist from childhood to adolescence and into adulthood [56].

Future research could use the DCE methodology specifically with CYP and incorporate statements of the COM-B to elucidate the contribution that capability and motivation make towards behaviour and its decision-making process. Embedding safety as a fourth attribute in the DCE would allow a comparison of this factor, which was not included in the current study. A more diverse range of methodologies, such as Q-methodology, combined with Geographic Information Systems (GIS) can provide insight into barriers and their location and further explore whether distance or rural/urban should be considered preferentially.

### 4.3. Conclusion

In conclusion, a discrete choice experimental method indicates that CYP and parents in Wales appear to have potential for behaviour change towards more active travel. Parents in particular base their decision making strongly on motivation, although actual behaviour might be more influenced by psychological capability. This highlights the complexity of promoting active travel. However, by using the COM-B model, it suggests that psychological capability should be considered in developing new interventions. One can build active travel routes (creating opportunity), but if people don’t perceive themselves to have the psychological capability or motivation, they may remain underused.

## Supporting information

S1 File Parent questionnaire. (PDF)

S2 File Children and young people questionnaire. (PDF)

S3 File Specification for the base case model. (PDF)

S4 File Data file. (XLSX)
