# Supplementary material for: Understanding the interactions that children and young people have with their natural and built environments: A survey to identify targets for active travel behaviour change in Wales
Source: PLoS One. 2024 Oct 18;19(10):e0311498. doi: 10.1371/journal.pone.0311498 (PMC11488727; doi:10.1371/journal.pone.0311498)
Supplement: S2 File — (PDF) [file pone.0311498.s002.pdf]

Holiadur Pobl Ifanc INHABIT / INHABIT Young People Questionnaire

Dewis iaith / Language Preference

Nodwch p'un a hoffech barhau yn Gymraeg neu yn Saesneg.
Please indicate whether you would like to continue in Welsh or English. ✱ *Required*

○ Welsh / Cymraeg ○ English / Saesneg

Participant Information Sheet

INHABIT - Understanding the INteractions that cHildren And young people have with their natural and Built envIronmenTs

Thank you for your interest in the study titled: '***INHABIT** - Understanding the **IN**teractions that c**H**ildren **A**nd young people have with their natural and **B**uilt env**I**ronmen**T**s*'.

Before you decide whether or not to take part, it is important for you to understand why the research is being undertaken and what it will involve. Please take time to read the following information carefully and discuss it with others including your family, if you wish.

1. What is the purpose of this study?

Spending time outdoors has been shown to support young peoples' good mental health and well-being. However, previous research suggests that young people are spending less time outdoors than at any other time in history. What we don't know however is whether this is the case in Wales. We also don't know whether young people think they have the capability, are motivated or have the opportunity to spend time outdoors. Therefore, this study aims to understand how young people in Wales are interacting with their built (human made surroundings) and natural (naturally occurring surroundings) environments, from both a parent's perspective and importantly from the perspective of young people. For the purposes of this study, we are interested in public outdoor spaces (not including private gardens).

2. Who is running the study?

The research team includes Dr Catherine Purcell (Cardiff University), Dr Emily Holmes (Bangor University), Dr Amy Mizen (Swansea University), Dr Tracie McKinney (University of South Wales), Dr Marco Arkesteijn (Aberystwyth University), Dr Kim Knowles (Aberystwyth University) and Dr Ginu Rajan (Cardiff Metropolitan University). In addition, members of the public, specifically four parents and three young people, are actively contributing to this study.

3. Who can take part?

You have been invited to take part because you live in Wales and you are aged between 12-16 years old.

4. Do I have to take part?

No, your participation in this research project is entirely voluntary and it is up to you to decide whether or not to take part. If you are unsure about whether to complete the questionnaire, then we suggest that you speak to your family about whether to take part or not. If you decide that you want to take part, then click the next button at the bottom of this information sheet to access the e-consent form, once you have provided your e-consent you will automatically access the questionnaire. If you decide not to take part, you do not have to explain your reasons and it will not affect your legal rights.

You are free to withdraw from the research project up to the point when you press the submit button. You do not have to explain your reasons for not completing and submitting the questionnaire. However, once you have submitted your questionnaire you will not be able to withdraw your data, this is because the questionnaire data is entirely anonymous so we will not be able to identify your specific questionnaire.

5. What will taking part involve?

If you do decide to take part, then click next at the end of this information, this will take you to an e-consent form to complete. If you complete the e-consent form, then when you click next you will be taken straight to the questionnaire.

The questionnaire should take no longer than 10 minutes to complete and is entirely anonymous. Once you have completed the questionnaire you will see a debrief sheet which provides you with contact details of organisations that you may want to access for support/information.

6. Will I be paid for taking part?

No. Unfortunately, you will not get paid for taking part in this study.

However, you do have the option to enter a prize draw: first prize is £25, second prize is £15 and third prize is £10, if you wish to enter the prize draw then please click on the link at the end of the questionnaire - you will need to ask a parent to enter their email address. Your parents email address will not be linked to your questionnaire responses. You can't provide your own email address. The prize draw will take place at the end of August 2023. Email addresses will not be linked to questionnaire data.

7. What are the possible benefits of taking part?

There may not be any direct benefit to you from taking part in this study. However, the findings from this study will enable us to better understand how young people in Wales are interacting with their built and natural environments, from a parent's perspective and importantly from the perspective of young people. In the longer term, this understanding may enable us to, for example, influence infrastructure changes that are evidence-based, such as location of cycle routes, or incentives for active travel.

8. What are the possible risks of taking part?

The questions ask you to consider your behaviour in relation to the outdoors, as well as what motivates you to spend time outdoors and whether you feel you have the capability and/or the opportunity to spend time outside. As such, the questions ask you to reflect on your interactions with outdoor space. This may, unintentionally, cause you some distress. In the event that you do experience any distress, please feel free to skip questions, or to exit the questionnaire entirely. If you exit the questionnaire before you have hit the submit button then your responses will not be saved.

At the end of the questionnaire you will see a debrief sheet, which provides you with contact details and websites of organisations that you may want to access for support/more information about how to access the outdoors. Once you have submitted your questionnaire your data will be entirely anonymous and stored securely (please see 'What will happen to my personal data' for more information).

9. Will my taking part in this research project be kept confidential?

All information collected from (or about) you during the research project will be kept confidential and any personal information you provide will be managed in accordance with data protection legislation. Please see 'What will happen to my Personal Data?' (below) for further information.

10. What will happen to my Personal Data?

Your parent's email address will be known if they agree to provide it in order for you to be included in the prize draw or if you email a member of the project team with a query. Once the prize draw has taken place (August 2023) and / or your query has been addressed, your email will be immediately deleted. All other data collected from the questionnaire however will be completely anonymous.

Cardiff University is the Data Controller and is committed to respecting and protecting your personal data in accordance with your expectations and Data Protection legislation. Further information about Data Protection, including:

- your rights
- the legal basis under which Cardiff University processes your personal data for research
- Cardiff University's Data Protection Policy
- how to contact the Cardiff University Data Protection Officer
- how to contact the Information Commissioner's Office

may be found at https://www.cardiff.ac.uk/public-information/policies-and-procedures/data-protection

Printed copies of the above-mentioned documentation and privacy notices can be provided on request.

11. What happens to the data at the end of the research project?

In accordance with the Cardiff University Records Retention Schedule (2021), your data (your anonymous questionnaire) will be destroyed after 5 years. It may however be published in support of the research project and/or retained indefinitely, where it is likely to have continuing value for research purposes. Your identity will not be added as the questionnaire is anonymous.

12. What will happen to the results of the research project?

It is our intention to publish the results of this research project in academic journals and present findings at conferences. We also plan to share the findings with parents and young people as well as youth organisations and other interested parties. Participants will not be identified in any report, publication or presentation. The results are likely to be published in 2024 and you will be able to obtain a copy of the published results by contacting any member of the research team.

13. What if there is a problem?

If you wish to complain or have grounds for concerns about any aspect of the manner in which you have been approached or treated during the course of this research, please contact Dr Catherine Purcell at PurcellC2@Cardiff.ac.uk. If you remain unhappy and wish to complain formally, you can do this by contacting the School of Healthcare Sciences Director of Research Governance (Dr Jen Davies daviesj@cardiff.ac.uk 02920688581).

If you are harmed by taking part in this research project, there are no special compensation arrangements. If you are harmed due to someone's negligence, you may have grounds for legal action, but you may have to pay for it.

14. Who is organising and funding this research project?

The research is funded by Welsh Crucible and organised by:

Dr Catherine Purcell
School of Healthcare Sciences
College of Biomedical and Life Sciences
Cardiff University
Tŷ Dewi Sant
Heath Park, Cardiff, CF14 4XN

Tel: +44 (0)29 2251 0961
Email: PurcellC2@Cardiff.ac.uk

Dr Emily Holmes
School of Medical and Health Sciences
Bangor University
Bangor
Gwynedd
LL57 2DG

Tel: +44(0) 1248 382709
Email: e.holmes@bangor.ac.uk

Dr Amy Mizen
Data Science, Health and Wellbeing
Swansea University
Singleton Park
Sketty
Swansea, SA2 8PP

Tel: +44(0) 1792 205678
Email: a.r.mizen@swansea.ac.uk

Dr Tracie McKinney
Faculty of Computing, Engineering and Science
University of South Wales
The Alfred Russel Wallace Building
Upper Glyntaff, Pontypridd, CF37 4BD

Tel: +44(0) 1443 4829433
Email: tracie.mckinney@southwales.ac.uk

Dr Marco Arkesteijn
Department of Life Sciences
Aberystwyth University
Carwyn James Building
Penglais
Aberystwyth, Ceredigion, SY23 3FD

Tel: +44(0) 1970 628559
Email: maa36@aber.ac.uk

Dr Kim Knowles
Department of Theatre, Film & Television
Aberystwyth University
Parry – Williams Building
Penglais
Aberystwyth, Ceredigion, SY23 3FL

Tel: +44(0) 1970 628700
Email: kik2@aber.ac.uk

Dr Ginu Rajan
Department of Applied Computing and Engineering
Cardiff Metropolitan University
Llandaff Campus
Western Avenue
Cardiff, CF5 2YB

Tel: +44(0)29 2041 2041
Email: GRajan@cardiffmet.ac.uk

15. Who has reviewed this research project?

This research project has been reviewed and given a favourable opinion by the School of Healthcare Sciences Research Ethics Committee, Cardiff University.

16. Further information and contact details

Should you have any questions relating to this research project, you may contact any member of the research team at the email addresses provided above. Any emails received by any member of the research team will be deleted as soon as queries have been responded to.

Thank you for considering taking part in this research project. Please feel free to download this participant information for your records by clicking here.

Dated 18.01.2023 version 2.0

e-Consent

School Research Ethics Committee (SREC) reference: REC985

Name of Principal Investigator: Dr Catherine Purcell

Contact email address: PurcellC2@cardiff.ac.uk

Cardiff University attaches high priority to the ethical conduct of research. We therefore ask you to consider the following points before completing this form. Completing this form confirms that you are happy to take part in the study.

The purpose of this e-consent form is to ensure that you are willing to take part in this study. If any of the statements are unclear please ask a parent to help you. You are free to withdraw by exiting the questionnaire at any time *without* penalty and your data will not be submitted.

I confirm that **I am aged between 12-16 years old.** ✱ *Required*

○ Yes ○ No

e-Consent

I confirm that **I have read the information sheet** dated 18.01.2023 version 2.0 for the above research project. ✱ *Required*

○ Yes ○ No

e-Consent

I confirm that **I have understood the information sheet** dated 18.01.2023 version 2.0 for the above research project and that I have had the opportunity to ask questions and that these have been answered satisfactorily. ✱ *Required*

○ Yes ○ No

e-Consent

I understand that my participation is voluntary and I am free to withdraw by exiting the questionnaire prior to pressing the submit button (I can stop and close the questionnaire before the end), but once I have submitted the questionnaire it will not be possible to withdraw. ✱ *Required*

○ Yes ○ No

e-Consent

I understand who will have access to information provided, how the data will be stored and what will happen to the data at the end of the research project. ✱ *Required*

○ Yes ○ No

e-Consent

I understand how the findings of the questionnaire will be used and that the results of the research project will be written up and published. ✱ *Required*

○ Yes ○ No

e-Consent

I understand that **if I decide to enter the prize draw that my parents email address must be provided** and this be stored securely until after the prize draw has taken place (August 2023). ✱ *Required*

○ Yes ○ No

e-Consent

I agree to take part in this research project. ✱ *Required*

○ Yes ○ No

Questionnaire

Following some brief questions about you, you are asked to respond to a series of questions relating to how you interact with your outdoor space. For the purposes of this questionnaire, we are defining outdoor space as any public space that is outside, **this doesn't include private gardens.**

Please select your age: ✱ *Required*

○ 12 years old ○ 13 years old ○ 14 years old
○ 15 years old ○ 16 years old

Please select your gender ✱ *Required*

What is your ethnic group? (Choose one option that best describes your ethnic group or background) ✱ *Required*

Other ethnic group

- ○ Arab
- ○ Other

If you selected Other, please specify:

Black / African / Caribbean / Black British

- ○ African
- ○ Caribbean
- ○ Other

If you selected Other, please specify:

Asian / Asian British

- ○ Indian
- ○ Pakistani
- ○ Bangladeshi
- ○ Chinese
- ○ Other

If you selected Other, please specify:

Mixed / Multiple ethnic groups

- ○ White and Black Caribbean
- ○ White and Black African
- ○ White and Asian
- ○ Other

If you selected Other, please specify:

White

- ○ English / Welsh / Scottish / Northern Irish / British
- ○ Irish
- ○ Traveller
- ○ Other

If you selected Other, please specify:

Which of the following characteristics best describes you (please select all that apply): ✱ *Required*

- ☐ I have no known additional needs
- ☐ I have communication and interaction needs
- ☐ I have cognitive (thinking) and additional learning needs
- ☐ I have social, emotional and mental health needs
- ☐ I have sensory and / or physical needs
- ☐ Prefer not to say

Other

Do you own a (please select all that apply): ✱ *Required*

- ☐ (Bi)cycle
- ☐ Scooter
- ☐ Skateboard
- ☐ e-Scooter
- ☐ e-Cycle
- ☐ None of these

Other

Please enter your home postcode (please enter the full postcode e.g. CF14 4XN, leaving a space between the two sections of the postcode). ***If you don't know your postcode please copy and paste this postcode CF14 4XN and then answer the next question instead.*** ✱ *Required*

Please enter a valid UK postcode.

If you don't know your postcode, please enter the name of the village/town/city that you live in.

What type of property do you live in? ✱ *Required*

- ○ House or bungalow with a private garden
- ○ House or bungalow with a shared garden
- ○ Flat, maisonette or apartment with a private garden
- ○ Flat, maisonette or apartment with a shared garden
- ○ A caravan or other mobile or temporary structure with a private garden
- ○ A caravan or other mobile or temporary structure with a shared garden
- ○ A property that has no access to a private or shared garden
- ○ House or bungalow on a farm
- ○ Other

If you selected Other, please specify:

On average, roughly how many hours do you spend on a screen (e.g. computer, phone) outside of school hours each week? ✱ *Required*

Please enter a number.

Please answer the following questions using a scale of 1 (strongly agree) to 5 (strongly disgaree)

I am physically able to access the outdoors ✱ *Required*

○ Strongly agree ○ Agree ○ Neither agree nor disagree
○ Disagree ○ Strongly disagree

I am physically able to spend time doing outdoor activities (e.g., cycling or pond dipping) ✱ *Required*

○ Strongly agree ○ Agree ○ Neither agree nor disagree
○ Disagree ○ Strongly disagree

I have the physical ability to independently access the outdoors ✱ *Required*

○ Strongly agree ○ Agree ○ Neither agree nor disagree
○ Disagree ○ Strongly disagree

I have the physical ability to access the outdoors safely ✱ *Required*

○ Strongly agree ○ Agree ○ Neither agree nor disagree
○ Disagree ○ Strongly disagree

My family are physically able to help me spend time outdoors ✱ *Required*

○ Strongly agree ○ Agree ○ Neither agree nor disagree
○ Disagree ○ Strongly disagree

I know why it is important to spend time outdoors ✱ *Required*

- ○ Strongly agree
- ○ Agree
- ○ Neither agree nor disagree
- ○ Disagree
- ○ Strongly disagree

I have lots of ideas of what I can do when I spend time outdoors ✱ *Required*

- ○ Strongly agree
- ○ Agree
- ○ Neither agree nor disagree
- ○ Disagree
- ○ Strongly disagree

My family believe that I have the psychological ability (i.e., I can engage in the necessary thought processes) to spend time outdoors ✱ *Required*

- ○ Strongly agree
- ○ Agree
- ○ Neither agree nor disagree
- ○ Disagree
- ○ Strongly disagree

I believe it is important for me to spend time outdoors ✱ *Required*

- ○ Strongly agree
- ○ Agree
- ○ Neither agree nor disagree
- ○ Disagree
- ○ Strongly disagree

My family makes time for outdoor activities ✱ *Required*

- ○ Strongly agree
- ○ Agree
- ○ Neither agree nor disagree
- ○ Disagree
- ○ Strongly disagree

I make time to be outdoors ✱ *Required*

- ○ Strongly agree
- ○ Agree
- ○ Neither agree nor disagree
- ○ Disagree
- ○ Strongly disagree

My family tries to make sure that I have enough time to spend outdoors ✱ *Required*

- ○ Strongly agree
- ○ Agree
- ○ Neither agree nor disagree
- ○ Disagree
- ○ Strongly disagree

I make plans to meet people outdoors ✱ *Required*

- ○ Strongly agree
- ○ Agree
- ○ Neither agree nor disagree
- ○ Disagree
- ○ Strongly disagree

My friends enjoy meeting up outdoors ✱ *Required*

- ○ Strongly agree
- ○ Agree
- ○ Neither agree nor disagree
- ○ Disagree
- ○ Strongly disagree

This is an attention check question please choose 'disagree' ✱ *Required*

- ○ Strongly agree
- ○ Agree
- ○ Neither agree nor disagree
- ○ Disagree
- ○ Strongly disagree

I generally prefer to spend time outdoors rather than indoors ✱ *Required*

- ◯ Strongly agree
- ◯ Agree
- ◯ Neither agree nor disagree
- ◯ Disagree
- ◯ Strongly disagree

Being outdoors makes me feel good ✱ *Required*

- ◯ Strongly agree
- ◯ Agree
- ◯ Neither agree nor disagree
- ◯ Disagree
- ◯ Strongly disagree

I want to spend time outdoors ✱ *Required*

- ◯ Strongly agree
- ◯ Agree
- ◯ Neither agree nor disagree
- ◯ Disagree
- ◯ Strongly disagree

I am motivated to spend time outdoors ✱ *Required*

- ◯ Strongly agree
- ◯ Agree
- ◯ Neither agree nor disagree
- ◯ Disagree
- ◯ Strongly disagree

As a family we enjoy spending time outdoors ✱ *Required*

- ◯ Strongly agree
- ◯ Agree
- ◯ Neither agree nor disagree
- ◯ Disagree
- ◯ Strongly disagree

The places that I have access to enables me to spend time outdoors ✱ *Required*

- ○ Strongly agree
- ○ Agree
- ○ Neither agree nor disagree
- ○ Disagree
- ○ Strongly disagree

I have experienced things in the environment (such as traffic) that stop me being outdoors ✱ *Required*

- ○ Strongly agree
- ○ Agree
- ○ Neither agree nor disagree
- ○ Disagree
- ○ Strongly disagree

There are local facilities that promote outdoor activities for me ✱ *Required*

- ○ Strongly agree
- ○ Agree
- ○ Neither agree nor disagree
- ○ Disagree
- ○ Strongly disagree

As a family we go to places that enable me to take part in outdoor activities ✱ *Required*

- ○ Strongly agree
- ○ Agree
- ○ Neither agree nor disagree
- ○ Disagree
- ○ Strongly disagree

We find that sometimes online questionnaires are taregeted by 'bots' which can make the data meaningless. Please select a fruit from the drop down list. ✱ *Required*

My friends encourage me to spend time outdoors ✱ *Required*

- ○ Strongly agree
- ○ Agree
- ○ Neither agree nor disagree
- ○ Disagree
- ○ Strongly disagree

My family encourages me to spend time outdoors ✱ *Required*

- ○ Strongly agree
- ○ Agree
- ○ Neither agree nor disagree
- ○ Disagree
- ○ Strongly disagree

I belong to organisations (e.g., Scouts, Guides etc) that enable me to take part in outdoor activities ✱ *Required*

- ○ Strongly agree
- ○ Agree
- ○ Neither agree nor disagree
- ○ Disagree
- ○ Strongly disagree

I would describe us as an 'outdoor family' ✱ *Required*

- ○ Strongly agree
- ○ Agree
- ○ Neither agree nor disagree
- ○ Disagree
- ○ Strongly disagree

I would spend more time outdoors if the benefits were immediate ✱ *Required*

- ○ Strongly agree
- ○ Agree
- ○ Neither agree nor disagree
- ○ Disagree
- ○ Strongly disagree

Please respond to the following questions by entering a number in the text box provided:

Please indicate roughly how many hours you spend outdoors in a normal week

	Number of hours per week during school term time ✱ *Required*	Number of hours per week during school holiday time ✱ *Required*
In winter		
In summer		

Please indicate roughly how many hours you spend outdoors doing activities that you have organised as a family to do together in a normal week

	Number of hours per week during school term time ✱ *Required*	Number of hours per week during school holiday time ✱ *Required*
In winter		
In summer		

Please indicate roughly how many hours you spend outdoors doing activities that have been organised by someone else in a normal week

	Number of hours per week during school term time ✱ *Required*	Number of hours per week during school holiday time ✱ *Required*
In winter		
In summer		

Please indicate roughly how many hours you spend outdoors doing activities that you have organised yourself and chosen to do in a normal week

	Number of hours per week during school term time ✱ *Required*	Number of hours per week during school holiday time ✱ *Required*
In winter		
In summer		

Finally, we’d like to ask you to answer some choice questions.

Imagine you have two options of how you make a journey. Please indicate which of the following options, Option A or Option B, you would prefer. There are no right or wrong answers, we are simply interested in your views.

	**Option A**	**Option B**
**Landscape**	**Rural** *Natural Surroundings*	**Residential** *Built Environment*
**Type of Travel**	**Active Travel** *e.g., Walk/Bicycle*	**Motorised Transport** *e.g., Car/Bus*
**Journey Time**	**10 minutes**	**25 minutes**

Which option would you prefer (please select A or B)? ✱ *Required*

○ Option A ○ Option B

	**Option A**	**Option B**
**Landscape**	**Residential** *Built Environment*	**Rural** *Natural Surroundings*
**Type of Travel**	**Active Travel** *e.g., Walk/Bicycle*	**Motorised Transport** *e.g., Car/Bus*
**Journey Time**	**25 minutes**	**10 minutes**

Which option would you prefer (please select A or B)? ✱ *Required*

○ Option A ○ Option B

	**Option A**	**Option B**
**Landscape**	**Rural** *Natural Surroundings*	**Residential** *Built Environment*
**Type of Travel**	**Motorised Transport** *e.g., Car/Bus*	**Active Travel** *e.g., Walk/Bicycle*
**Journey Time**	**25 minutes**	**10 minutes**

Which option would you prefer (please select A or B)? ✱ *Required*

◯ Option A ◯ Option B

	Option A	Option B
Landscape	**Residential** *Built Environment*	**Rural** *Natural Surroundings*
Type of Travel	**Motorised Transport** *e.g., Car/Bus*	**Active Travel** *e.g., Walk/Bicycle*
Journey Time	**10 minutes**	**25 minutes**

Which option would you prefer (please select A or B)? ✱ *Required*

◯ Option A ◯ Option B

Debrief Page

Thank you for taking part in this study.

What were we looking for?

As mentioned in the Participant Information Sheet, this study aims to understand how young people in Wales are interacting with their built and natural environment, from a parent's perspective and importantly from the perspective of young people.

Storage of Data

As outlined in the Participant Information Sheet, the data you provide is processed in compliance with GDPR. All data will remain completely confidential and anonymous.

What should I do if I have concerns about this study?

If you wish to complain or have grounds for concerns about any aspect of the manner in which you have been approached or treated during the course of this research, please contact Dr Catherine Purcell at PurcellC2@Cardiff.ac.uk. If you remain unhappy and wish to complain formally, you can do this by contacting the School of Healthcare Sciences Director of Research Governance (Dr Jen Davies daviesj@cardiff.ac.uk 02920688581).

Support and Information

If any aspect of this study has inadvertently caused you any distress please contact your GP. If you wish to raise any safeguarding concerns, please contact your school's Safeguarding Officer.

The following organisations may be of interest and may be able to offer advice and opportunities for you to spend time outdoors:

- National Trust Wales - https://www.nationaltrust.org.uk/visit/wales
- Woodland Trust - https://www.woodlandtrust.org.uk/
- RSPB - https://www.rspb.org.uk/
- Girlguiding Cymru - https://girlguidingcymru.org.uk/
- Scouts Cymru - https://scoutscymru.org.uk/
- Wood Craft Folk - https://woodcraft.org.uk/
- Wales Federation of Young Farmers - https://yfc.wales/
- Play Wales - https://www.playwales.org.uk/eng/
- Urdd Gobaith Cymru - https://www.urdd.cymru/cy/
- Disability Sports Wales - https://www.disabilitysportwales.com/en-gb#cookieConsent
- National Parks Wales - https://www.nationalparkswales.uk/npw
- Children's Commissioner for Wales - https://www.childcomwales.org.uk/
- Playful Childhoods - https://www.playfulchildhoods.wales/Listing/Category/supporting-teenagers

Final page

Thank you for completing this questionnaire, if you would like to enter the prize draw please click here and ask a parent to complete the two questions.

Key for selection options

11 - Please select your gender
- Male
- Female
- Other
- Prefer not to say

12 - What is your ethnic group? (Choose one option that best describes your ethnic group or background)
- White
- Mixed / Multiple ethnic groups
- Asian / Asian British
- Black / African / Caribbean / Black British
- Other ethnic group
- Prefer not to say

42 - We find that sometimes online questionnaires are tareqeted by 'bots' which can make the data meaningless. Please select a fruit from the drop down list.
- iPad
- Lawnmower
- Banana
- Pigeon
