# Supplementary material for: Understanding the interactions that children and young people have with their natural and built environments: A survey to identify targets for active travel behaviour change in Wales
Source: PLoS One. 2024 Oct 18;19(10):e0311498. doi: 10.1371/journal.pone.0311498 (PMC11488727; doi:10.1371/journal.pone.0311498)
Supplement: S3 File — (PDF) [file pone.0311498.s003.pdf]

Understanding the intersections that children and young people have with their natural and built environments

SUPPORTING INFORMATION: FILE 3

Annex 1: The base case model was specified as:

$U_i = \beta_0 + \beta_1$ (landscape_resdiential) + β_2 (landscape_rural) + β_3 (travel_active) + β_4 (travel_motorised) + β_5 (time) + ε

Where:

U_i= utility derived by individual

β_0= constant term

β_i= estimated coefficient for each attribute (variable)

ε= error term

Marginal rates of substitution of journey time for landscape and travel type were calculated as:

$MRS_{time} = -\beta_i / \beta_{time}$

The expected 'utility' of four alternative journey options was calculated by weighting and summing the coefficients (β) from the respective regression models:

$Utility = \Sigma(\beta_i * JP_i)$

Where ji is the journal parameter of the associated coefficient, and the expected probability of uptake is derived as:

$P = \exp(\text{utility } J_i)/\Sigma\exp(J_j)$.
